# Left ventricular concentric remodeling is highly common among veterans deployed to Southwest Asia and is associated with impaired exercise performance

**DOI:** 10.14814/phy2.70445

**Published:** 2025-07-09

**Authors:** Steven J. Cassady, Thomas J. Abitante, Gregory G. Pappas, Thomas Alexander, Michael J. Falvo, Post‐Deployment Cardiopulmonary Evaluation Network, Mehrdad Arjomandi, Danielle Glick, Silpa Krefft, John Osterholzer, Bradley Richmond, Anays Sotolongo

**Affiliations:** ^1^ Baltimore VA Medical Center VA Maryland Health Care System Baltimore Maryland USA; ^2^ Division of Pulmonary and Critical Care Medicine, Department of Medicine University of Maryland School of Medicine Baltimore Maryland USA; ^3^ Airborne Hazards and Burn Pits Center of Excellence VA New Jersey Health Care System East Orange New Jersey USA; ^4^ New Jersey Medical School Rutgers University Newark New Jersey USA

**Keywords:** cardiopulmonary exercise test, left ventricular remodeling, military exposures

## Abstract

Left ventricular concentric remodeling (LVCR), a pathological process of adaptive myocardial change, may be a precursor state for systolic and diastolic dysfunction and LV hypertrophy. Potential cardiotoxic airborne hazards present in combat zones in excess may increase the risk for adverse cardiac remodeling in deployed veterans. One hundred and thirty‐nine deployed veterans were included in this study and underwent evaluations including transthoracic echocardiography and cardiopulmonary exercise testing (CPET). Relative wall thickness was used to classify LV geometry as normal, concentric or eccentric hypertrophy, or LVCR. Observed rates of LVCR were compared to those reported in the Framingham Heart Study (FHS), and CPET results were compared between those with and without LVCR. Rates of LVCR in our veteran sample (12%–34%) were elevated compared to FHS (8%–21%), despite similar demographics and risk factors. Veterans with LVCR had reduced exercise capacity, greater ventilatory inefficiency, and increased heart rate reserve relative to those without. Deployment length was associated with increased odds of having LVCR (aOR = 1.04, 95% CI [1.01, 1.07]). Abnormal LV geometry, specifically LVCR, was common in our veteran sample and exceeded historical civilian rates. The presence of LVCR was associated with impaired exercise performance and was more likely in those with longer deployment lengths.

## 1 BACKGROUND

More than three million United States military personnel have been deployed in conflicts in the Southwest Asia Theater of Military Operations since 2001. A variety of symptoms have been described by returning veterans, particularly dyspnea, resulting in a broad effort to investigate and categorize the diagnoses and mechanisms underpinning this symptom in this cohort (Krefft et al., 2020). A major concern is the effect of wartime airborne hazards on long‐term cardiopulmonary health among exposed veterans. This concern appears justified in light of fine particulate matter (PM_2.5_) air pollution levels (>100 μg/m^3^) that far exceed military exposure guidelines (65 μg/m^3^ for 24‐h exposure, 15 μg/m^3^ daily average for 1 year), owing to dust storms, local ambient air pollution, and smoke from open air burn pits (Engelbrecht, McDonald, Gillies, Jayanty, et al., 2009). Soldiers with longer deployments to military bases with open air burn pits are at demonstrably increased risk of respiratory and cardiovascular conditions, including hypertension (Savitz et al., 2024). Given this increased exposure‐mediated risk, veterans deployed to these regions may be at increased risk for cardiac remodeling, possibly compounded by an increased prevalence of obesity in the veteran population in general (Breland et al., 2017).

Left ventricular concentric remodeling (LVCR) is an adaptive response by the myocardium to normalize wall stress brought on by increased cardiac afterload (Rosen et al., 2005). This process is reflected by an increase in relative wall thickness (RWT) without a concomitant change in left ventricular mass and carries an elevated risk of developing myocardial dysfunction and systolic and diastolic heart failure, especially amongst hypertensive individuals (Nauta et al., 2020). However, altered myocardial geometry, including LVCR, is also found more frequently in normotensive individuals with obesity and/or type 2 diabetes mellitus, suggesting an effect of altered metabolism on the development of these changes. Environmental risk factors such as air pollution, already shown to have a causal relationship with cardiovascular morbidity and mortality, have also been shown to have a variety of cardiotoxic effects, including the development of pathological ventricular remodeling (Brook et al., 2010). Specific mechanisms driving this process may include systemic inflammation, oxidative stress, autonomic nervous system imbalance, and direct cardiotoxicity (Liu et al., 2015).

To our knowledge, assessment of cardiac geometry in deployed veterans has not been systematically investigated. The primary purpose of our study was to investigate the prevalence of LVCR in two cohorts of deployed veterans with reported airborne hazards exposure and to explore the association between altered cardiac geometry and cardiopulmonary function.

## 2 METHODS

### 2.1 Study participants

A total of 139 veterans deployed to the Southwest Asia Theater of Military Operations (predominantly post‐9/11) comprised our analytical sample. All veterans were either participating in a specialty clinical evaluation program (*n* = 92) (Davis et al., 2022; Lange et al., 2013) at one of six VA Medical Centers (Ann Arbor, MI; Baltimore, MD; Denver, CO; Nashville, TN; East Orange, NJ; San Francisco, CA) or volunteering for a clinical research study at the East Orange, NJ VA Medical Center (*n* = 47). Both cohorts underwent comprehensive, multiday assessments that included transthoracic echocardiography, assessment of cardiopulmonary function, symptom assessments, and review of medical and military histories. Per our research protocol's enrollment criteria, all 47 volunteers were required to be free of any confirmed or self‐reported cardiovascular or respiratory diagnosis at the time of the study. Study procedures were approved and under the oversight of the VA New Jersey Health Care System's Institutional Review Board and Research and Development Committee (#1577315, 1577279). All subjects provided written consent prior to participating.

All data were abstracted from clinical and research records that included questionnaires, medical histories, and in‐laboratory assessments. Veterans were classified into normal, overweight, and obese body mass index (BMI) categories—that is, <25 kg/m^2^, ≥25 kg/m^2^ to <30 kg/m^2^, or ≥30 kg/m^2^. The presence or absence of comorbidities of hypertension, diabetes mellitus, and obstructive sleep apnea were confirmed on medical chart review. We further defined the presence of hypertension based upon self‐report (i.e., those without a local medical record), resting systolic blood pressure ≥140 mmHg, or diastolic blood pressure ≥ 90 mmHg at the time of visit, or current use of an antihypertensive medication (i.e., angiotensin‐converting enzyme inhibitor, angiotensin receptor blocker, beta blockers, calcium channel blocker, or thiazide diuretics).

Health status and symptoms were also assessed via standardized questionnaires including: (1) the Sino‐Nasal Outcome Test (SNOT‐22) (Hopkins et al., 2009), (2) the Modified Medical Research Council dyspnea scale (mMRC) (Fletcher et al., 1959), (3) the Reflux Disease Questionnaire (RDQ) (Shaw et al., 2001), (4) the Generalized Anxiety Disorder 7 (GAD‐7) questionnaire (Spitzer et al., 2006), and (5) the Patient Health Questionnaire 9 (PHQ‐9) (Kroenke et al., 2001). The presence of probable PTSD was also assessed by either the Primary Care PTSD screen for DSM‐5 (PC‐PTSD‐5) (Prins et al., 2015) in the specialty clinical evaluation veterans or by the PTSD Checklist for DSM‐5 (PCL‐5) (Weathers et al., 2013) in the research volunteer veterans. Also included was a functional activity score, an average of five questions where individuals report the difficulty in performing five basic physical tasks (National Academies of Sciences, Engineering, and Medicine, et al., 2017).

### 2.2 Transthoracic echocardiography and left ventricular geometry

All participants underwent resting transthoracic echocardiography (TTE) in accordance with published guidelines (Mitchell et al., 2019). The primary echocardiographic parameters of interest were manually extracted from the clinical report in the electronic health records and included two‐dimensional measurements of left ventricular dimensions: left ventricular end‐diastolic diameter (LVEDD), posterior wall thickness at end‐diastole (PWd), and interventricular septal wall thickness at end‐diastole (IVSd), as well as height, weight, and sex to calculate body surface area. We determined the presence and phenotype of left ventricular remodeling by calculating relative wall thickness (RWT) and left ventricular mass index (LVMI). RWT was calculated by the equation (IVSd + PWd)/LVEDD (Yamaguchi et al., 2019). A threshold of >0.42 was used to determine elevated RWT (Lang et al., 2015). We estimated LVMI (g/m^2^) as left ventricular mass calculated by Devereux's method (Devereux, 1987) divided by the body surface area. Left ventricular concentric remodeling (LVCR) is defined as a RWT >0.42 and LVMI ≤95 g/m^2^ (women) or ≤115 g/m^2^ (men). Concentric and eccentric hypertrophy were defined as LVMI >95 g/m^2^ (women) or >115 g/m^2^ (men) with a RWT >0.42 or ≤0.42, respectively. Rates of LVCR in our population were compared to those reported in the Framingham Heart Study cohort of civilians (von Jeinsen et al., 2020). Additional parameters of interest focused on diastolic function assessment included lateral E/e', mitral valve E/A ratio, left atrial volume index (LAVI), and estimated right ventricular systolic pressure (RVSP) and were also extracted from clinical records.

### 2.3 Military history and exposures

Military and exposure history were assessed as the total deployment length (cumulative days across all military deployments), time since returning from last deployment, time since first exposure (first day of first deployment), and select exposure endorsements and scores. Deployment lengths were estimated by aggregating the total days of all self‐reported land‐based deployments to the Southwest Asia Theater of Military Operations after 9/11/01. All veterans provided a binary self‐report endorsement of deployment‐related exposures including burn pits, sand/dust storms, vehicle exhaust, local industrial pollution, and blast exposure. Veterans undergoing specialty clinical evaluation also complete a more comprehensive exposure assessment developed by the VA's Cooperative Studies Program #595, Service and Health Among Deployed Veterans (SHADE) study as previously described by Garshick et al. (2024) Details of the SHADE study and results in our veteran cohort are included in the Appendix S1.

### 2.4 Cardiopulmonary exercise testing and pulmonary function testing

All veterans underwent a standardized symptom‐limited maximal cardiopulmonary exercise test (CPET) using a motor‐driven treadmill (Bruce or modified Bruce protocol; *n* = 28) or cycle ergometer (25 W/min; *n* = 84) (American Thoracic Society & American College of Chest Physicians, 2003). All CPETs were assessed for valid effort as defined as a measured peak heart rate (HR) >85% of age‐predicted maximum or a respiratory exchange ratio (RER) >1.0. Oxygen consumption (V̇O_2_), carbon dioxide production (V̇CO_2_), ventilation (V̇_E_), HR, and O_2_ pulse (V̇O_2_/HR) were obtained breath‐by‐breath during exercise. Blood pressure was measured every 2 min during exercise. Ventilatory efficiency was defined by the V̇_E_/V̇CO_2_ nadir, and the chronotropic response to exercise was calculated per the Wilkoff and Miller method (Wilkoff & Miller, 1992). Select CPET variables were expressed in absolute values and relative to predicted normal values (De Souza E Silva et al., 2018). Due to the variation in protocols, only values at peak exercise (average of the last 30 s of exercise) were utilized for a comparison between the veterans with and without LVCR. On a separate day, complete pulmonary function testing (PFT) was performed in accordance with published standards (Stanojevic et al., 2022) using commercially available equipment. PFT variables of interest included those from spirometry—forced expiratory volume in 1 s (FEV_1_), forced vital capacity (FVC), and FEV_1_/FVC ratio; body plethysmography—total lung volume (TLC), residual volume (RV), and RV/TLC ratio; and diffusion capacity for carbon monoxide (DL_CO_). These values were expressed both as absolute values and as percent of predicted (Quanjer et al., 2012).

### 2.5 Statistical analysis

Rates of abnormal LV geometry were calculated for the total cohort, within BMI classes, and among veterans with hypertension or diabetes mellitus. These rates were compared to a published historical sample (Framingham Heart Study) (von Jeinsen et al., 2020). To examine differences in our sample characteristics that may influence LV geometry and cardiopulmonary outcomes, veterans of the first cohort who sought treatment in specialized evaluation programs were compared to nontreatment seeking veteran volunteers of the second cohort. Subsequent analyses (demographics and cardiopulmonary) were pooled across all veterans. Given the small numbers of participants with concentric or eccentric hypertrophy (*n* = 5), further analysis beyond quantification of abnormal LV geometry was restricted to veterans with LVCR or normal geometry.

Group comparisons were conducted as a function of cohort (i.e., specialty clinical evaluation vs. research study volunteer) and cardiac geometry (i.e., normal vs. LVCR [pooled veteran sample]). Normality was assessed using the Shapiro–Wilk test. For continuous variables, *T*‐test, or Wilcoxon rank‐sum test were used as appropriate, with effect sizes reported using Cohen's d with Hedges' correction. Chi‐squared tests and Cramer's V were used for categorical variables. Statistical significance was set at *p* < 0.05.

Multivariate linear regression models were used to assess the relationship between RWT and select CPET parameters at peak exercise, adjusting for other variables with a known effect on exercise performance (age, BMI, sex, and CPET modality), as well as the presence of hypertension. Total deployment lengths were also included in the models as a proxy for duration of airborne hazard exposure. Additional models were used to estimate the adjusted differences in CPET outcomes between Veterans with and without LVCR. Adjusted associations are presented as beta‐coefficients and corresponding 95% confidence intervals and *p* values. Lastly, logistical regression models were performed to assess the influence of total deployment length and the five SHADE exposure domains on LV geometry, adjusting for known risk factors for concentric remodeling. All statistical analyses were performed using R Studio.

## 3 RESULTS

### 3.1 Sample description

Baseline characteristics of our sample are reported in Table 1 for the entire sample and separately for cohort type (i.e., specialty clinical evaluation vs. research study volunteer). Veterans undergoing specialty evaluation were older, had more comorbidities, had increased lateral E/e', E/A, and right ventricular systolic pressure values, and reported more symptoms than veterans who volunteered for a research study. Research study volunteers more frequently self‐identified as Hispanic or Latino and had fewer symptoms of anxiety and depression than those undergoing specialty evaluation. Demographic details of our sample and the Framingham Heart Study can be found in Table S1. Relative to the Framingham Heart Study population used for comparison of LVCR prevalence, our combined veteran sample (*n* = 139) had similar smoking histories and rates of hypertension, but was younger, composed of more men, was more overweight and obese, and was less frequently of European ancestry. Only one veteran had diabetes mellitus, thereby preventing meaningful comparison.

**TABLE 1:** Demographic characteristics, medical histories, and military histories of study participants, stratified by participant cohort.

	Combined veteran cohort (*n* = 139)		Research study volunteer (*n* = 47)		Specialty clinical evaluation (*n* = 92)		*p* Value	Effect size
	*n*	Mean (SD)	*n*	Mean (SD)	*n*	Mean (SD)		
Age	139	44.27 (9.35)	47	40.36 (7.78)	92	46.27 (9.49)	<0.001*	0.68
Sex (female)	19	13.67%	5	10.64%	14	15.22%	0.63	0.04
Race	136		47		89		0.20	0.19
White	110	80.88%	41	87.23%	69	77.53%		
Black	15	11.03%	5	10.64%	10	11.24%		
Asian	3	2.21%	1	2.13%	2	2.25%		
Other/multiple	8	5.88%	0	0.00%	8	8.99%		
Ethnicity	125		46		79			
Non Hispanic or Latino	88	70.40%	16	34.78%	72	91.14%	<0.001*	0.58
Hispanic or Latino	37	29.60%	30	65.22%	7	8.86%		
BMI	139	30.39 (5.33)	47	28.32 (3.44)	92	31.45 (5.81)	0.001*	0.65
Normal	17	12.23%	9	19.15%	8	8.70%	0.008*	0.26
Overweight	62	44.60%	26	55.32%	36	39.13%		
Obese	60	43.17%	12	25.53%	48	52.17%		
HTN (% yes)	139	27.34%	47	8.51%	92	36.96%	<0.001*	0.29
OSA (% yes)	139	43.88%	47	0.00%	92	66.30%	<0.001*	0.62
Smoking	129		47		82			
Current	2	1.55%	1	2.13%	1	1.22%	0.87	0.05
Former	41	31.78%	14	29.79%	27	32.93%		
Never	86	66.67%	32	68.09%	54	65.85%		
Pack years	43	5.34 (5.72)	15	3.88 (3.44)	28	5.71 (6.15)	0.42	0.32
Resp symptoms								
Shortness of breath (% yes)	131	67.18%	47	17.02%	84	95.24%	<0.001*	0.78
Sputum (% yes)	131	54.20%	47	23.40%	84	71.43%	<0.001*	0.45
Cough (% yes)	132	46.97%	47	8.51%	85	68.24%	<0.001*	0.56
Questionnaires								
SNOT22	130	33.68 (21.9)	47	20.17 (16.53)	83	41.33 (20.92)	<0.001*	1.12
MMRC	117	1.17 (0.95)	36	0.47 (0.61)	81	1.48 (0.91)	<0.001*	1.30
Functional score^a^	130	2.22 (0.96)	47	1.57 (0.68)	83	2.59 (0.90)	<0.001*	1.28
RDQ	132	4.51 (4.95)	47	1.47 (2.57)	85	6.18 (5.16)	<0.001*	1.15
GAD7	115	7.06 (6.28)	47	4.96 (6.11)	68	8.52 (6.02)	<0.001*	0.58
PHQ‐9	77	8.16 (6.14)	12	5.42 (6.14)	67	8.66 (6.05)	0.04*	0.52
PTSD^b^ (% yes)	81	20.99%	12	25.00%	69	20.29%	>0.9	0.00
Military history								
Total deploy length (Days)	135	496.50 (375.47)	47	535.57 (375.38)	88	475.63 (376.00)	0.18	016
Months since last deploy	135	154.38 (69.16)	47	141.32 (50.10)	88	161.36 (76.81)	0.22	0.31
Months since first exposure	135	203.47 (79.31)	47	173.61 (42.33)	88	219.41 (89.45)	0.002*	0.65
Exposures								
Blast	137	49.64%	47	68.09%	90	40.00%	0.002*	0.30
Dust storms	128	92.19%	47	85.11%	81	96.30%	0.01*	0.27
Burn pits	128	68.75%	47	80.85%	81	61.73%	0.001*	0.32
Vehicle exhaust	128	79.69%	47	72.34%	81	83.95%	0.18	0.12
Cardiac measures								
RWT	139	0.39 (0.10)	47	0.36 (0.10)	92	0.40 (0.09)	0.03*	0.43
LVMI (g/m^2^)	139	70.93 (19.52)	47	69.57 (20.29)	92	71.63 (19.18)	0.67	0.10
LAVI (mL/m^2^)	75	21.79 (5.87)	47	20.77 (4.88)	28	23.52 (6.99)	0.08*	0.46
E/A Ratio	135	1.24 (0.47)	47	1.33 (0.31)	88	1.88 (0.47)	0.003*	0.37
Lateral E/e'	109	6.47 (2.43)	47	5.80 (1.52)	62	6.98 (2.84)	0.04*	0.52
RVSP (mmHg)	66	21.95 (6.86)	41	20.06 (6.06)	25	25.01 (7.09)	0.002*	0.75
Resting systolic BP (mmHg)	99	120.02 (12.78)	39	114.59 (12.16))	58	123.20 (11.75)	<0.001*	0.72
Resting diastolic BP (mmHg)	99	80.95 (13.94)	39	73.23 (9.17)	58	86.76 (13.8)^a^	<0.001*	1.14

*Note*: Unadjusted analyses (*p* values and effect sizes) between the Research Study Volunteers and Specialty Clinical Evaluations were conducted with *t*‐tests and Wilcoxon signed‐rank tests (as required) and Cohen's *d* with Hedges correction for continuous variables and with chi‐squared and Cramer's V for categorical variables, respectively. Data are reported as mean (standard deviation) or proportion (%).

^a^ Mean score of 5 questions rating the difficulty of basic daily activities (e.g., walk 1 mile, walk up steep hill, and 12 stairs). Answers range from 0 (no difficulty) to 5 (incapable).

^b^ Probable PTSD determined based on the DSM‐5 criteria for the Primary Care PTSD Screen for DSM‐5 (Specialty Clinical Evaluation) or the PTSD Checklist for DSM‐5 (Research Volunteers).

* Denotes statistical significance (*p* < 0.05).

### 3.2 Transthoracic echocardiography and left ventricular geometry

**FIGURE 1:**
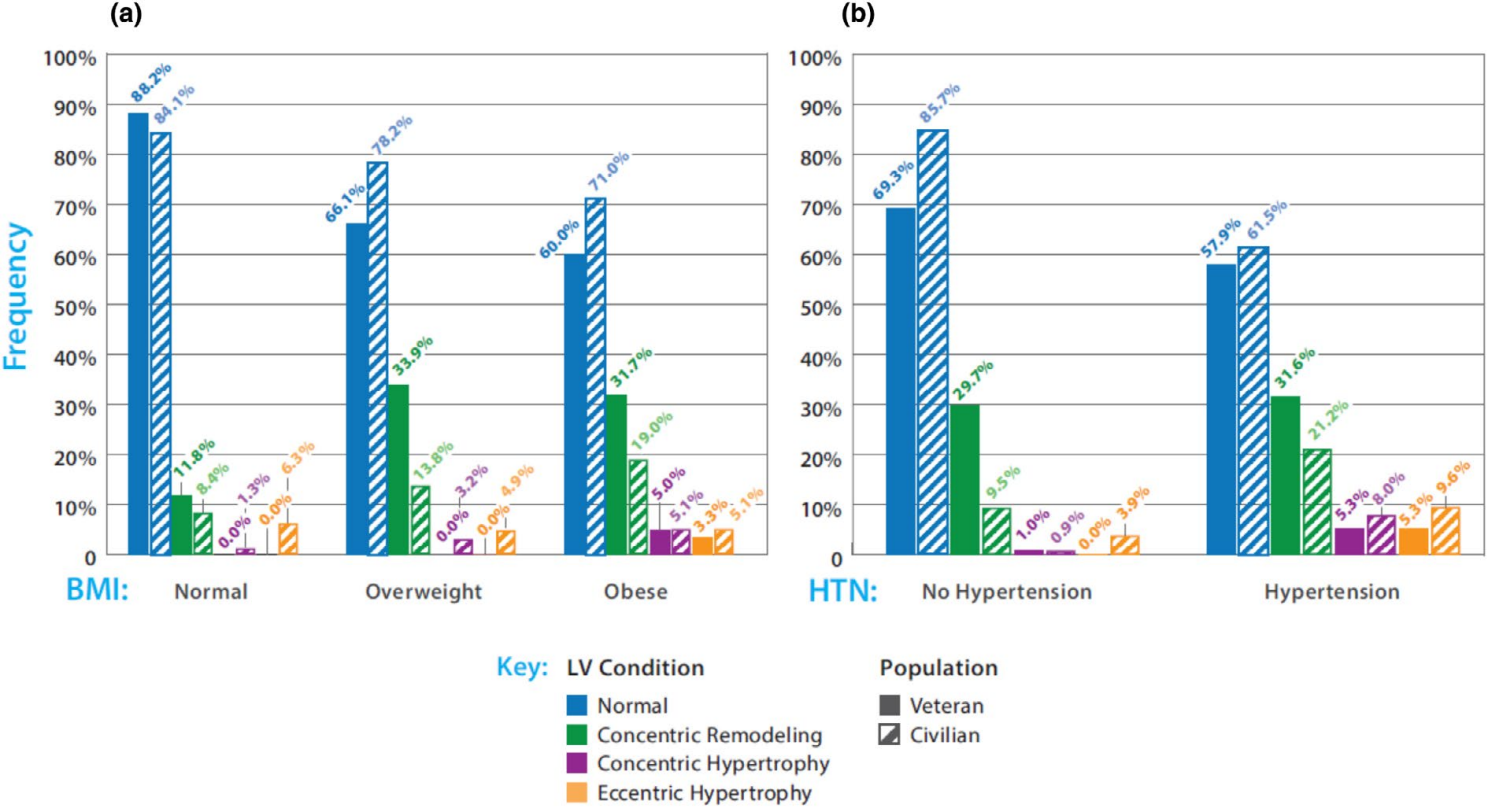
Frequency of LV concentric remodeling in our deployed veteran sample (*n* = 139) compared to a historical civilian population. (a) Body mass index (BMI); percentage of those within a particular BMI group (normal, overweight, and obese), regardless of hypertension status, having each LV condition, stratified by population. (b) Presence of hypertension (HTN); percentage of those within each HTN category (No/Yes), regardless of BMI group, having each LV condition, stratified by population. Historical civilian population rates were extrapolated from Von Jenison et al. 2020 (*n* = 5432), The civilian sample extrapolation excluded subjects with diabetes.

Abnormal LV geometry was present in 33.8% (47 of 139) of our sample and was predominantly LVCR (42 of 47). The prevalence of LVCR is depicted in Figure 1, with comparison to previously published rates derived from the historical cohort (von Jeinsen et al., 2020). Frequencies of LVCR were similar between our cohorts (specialty evaluation: 31.5%, research volunteers: 27.7%). In the combined group, 12% of patients with a normal BMI had LVCR, compared to 8% in the historical cohort. Overweight patients had a prevalence of 34% versus 14% in the control cohort, and obese patients 32% versus 19%. When divided based on the presence of comorbidities, rates in the combined cohort were roughly equal, with 32% and 30% of those with or without hypertension having LVCR, respectively. Both rates are higher than those in the historical cohort, in which rates were 21% and 10% with and without hypertension, respectively.

One hundred and thirty‐four veterans were determined to have LV geometry classified either as normal or LVCR. Descriptive statistics for veterans with and without LVCR are shown in Table 2. Three and two veterans were classified as concentric hypertrophy and eccentric hypertrophy, respectively, based on LVMI calculation, and were not included in subsequent analyses. Notably, 10 veterans undergoing specialty evaluation were described on physician read of their echocardiogram as having at least mild left ventricular hypertrophy (LVH) by visual assessment, and two veterans were read as having moderate or severe reductions in left ventricular ejection fraction. Veterans in the LVCR group were slightly older and had slightly higher rates of overweight and obesity, though BMI was not significantly different between the groups (Table 2). Systolic and diastolic blood pressures, LVMI, lateral E/e', mitral valve E/A ratio, LAVI, and estimated right ventricular systolic pressure were similar between groups.

**TABLE 2:** Demographic characteristics, medical and military histories of study participants stratified by left ventricular geometry, as determined by a resting transthoracic echocardiogram.

	Normal (*n* = 92)		LVCR (*n* = 42)		*p* Value	Effect size
	*n*	Mean	*n*	Mean		
% Treatment seeking	59	64.13%	29	69.05%	0.72	0.03
Age	92	43.48 (9.18)	42	45.10 (9.72)	0.44	0.17
Sex (female)	10	10.87%	9	21.43%	0.17	0.12
Race	90		41		0.98	0.04
White	73	81.11%	32	78.05%		
Black	10	11.11%	5	12.20%		
Asian	2	2.22%	1	2.44%		
Other/multiple	5	5.56%	3	7.32%		
Ethnicity	88		37			
Non Hispanic or Latino	64	72.73%	24	64.86%	0.51	0.06
Hispanic or Latino	24	27.27%	13	35.14%		
BMI	92	29.73 (4.81)	42	30.77 (4.94)	0.19	0.21
Normal	15	16.30%	2	4.76%	0.18	0.16
Overweight	41	44.57%	21	50.00%		
Obese	36	39.13%	19	45.24%		
HTN (% yes)	92	23.91%	42	28.57%	0.72	0.03
OSA (% yes)	92	40.22%	42	47.62%	0.54	0.05
Smoking	87		38			
Current	2	2.30%	0	0.00%	0.64	0.09
Former	28	32.18%	13	34.21%		
Never	57	65.52%	25	65.79%		
Pack years	26	4.58 (4.19)	10	7.25 (8.43)	0.17	0.47
Resp symptoms						
Shortness of breath (% yes)	88	62.50%	39	76.92%	0.17	0.12
Sputum (% yes)	88	53.41%	39	56.41%	0.90	0.01
Cough (% yes)	89	44.94%	39	51.28%	0.64	0.04
Questionnaires						
SNOT2	88	34.02 (21.52)	38	32.74 (22.48)	0.74	0.06
MMRC	79	1.08 (0.96)	34	1.32 (0.88)	0.23	0.25
Functional score^a^	88	2.13 (0.97)	38	2.36 (0.88)	0.15	0.25
RDQ	89	4.97 (5.23)	39	3.69 (4.25)	0.32	0.27
GAD7	76	7.12 (6.37)	35	7.06 (6.19)	0.95	0.01
PHQ‐9	51	9.10 (6.42)	24	6.19 (6.88)	0.16	0.37
PTSD^b^ (% yes)	52	26.92%	25	12.00%	0.24	0.13
Military history						
Total deploy length (Days)	89	449.40 (343.22)	41	614.01 (434.26)	0.009*	0.42
Months since last deploy	89	156.08 (66.70)	41	142.48 (67.12)	0.17	0.20
Months since first exposure	89	193.51 (71.24)	41	208.75 (79.60)	0.39	0.20
Exposures						
Blast	92	51.09%	41	46.34%	0.30	0.13
Dust storms	84	92.86%	40	92.50%	0.63	0.09
Burn pits	84	70.24%	40	65.00%	0.84	0.05
Vehicle exhaust	84	79.76%	40	77.50%	0.96	0.01
Cardiac measures						
RWT	92	0.34 (0.06)	42	0.49 (0.07)	<0.001*	2.46
LVMI (g/m^2^)	92	67.84 (16.79)	42	70.68 (14.34)	0.32	0.18
LAVI (mL/m^2^)	50	20.95 (5.67)	23	23.39 (6.24)	0.08	0.40
E/A ratio	89	1.26 (0.47)	41	1.21 (0.34)	0.79	0.11
Lateral E/e'	74	6.24 (2.15)	32	6.61 (2.61)	0.46	0.15
RVSP (mmHg)	47	21.68 (6.47)	19	23.37 (7.24)	0.38	0.43
Resting systolic BP (mmHg)	69	119.19 (13.04)	29	121.12 (11.51)	0.44	0.15
Resting diastolic BP (mmHg)	69	81.10 (19.93)	29	81.86 (13.80)	0.70	0.05

*Note*: Participants with either concentric or eccentric hypertrophy (*n* = 5) were excluded. *p* values and effect sizes were determined by *t*‐tests or Wilcoxon signed‐rank tests and Cohen's *d* with Hedges correction for continuous variables and with chi‐squared and Cramer's V for categorical variables, respectively. Values listed as either mean (standard deviation) or proportion (%).

^a^ Mean score of 5 questions rating the difficulty of basic daily activities (e.g., walk 1 mile, walk up steep hill, and 12 stairs). Answers range from 0 (no difficulty) to 5 (incapable).

^b^ Probable PTSD determined based on the DSM‐5 criteria for the Primary Care PTSD Screen for DSM‐5 (Specialty Clinical Evaluation) or the PTSD Checklist for DSM‐5 (Research Volunteers).

* Denotes statistical significance (*p* < 0.05).

### 3.3 Military exposures

Deployment length and time since last deployment between research volunteer and specialty clinical evaluation veteran cohorts were similar, and despite self‐report exposure histories differing, they were uniformly elevated in both cohorts (Table 1). When comparing those with normal geometry versus LVCR, self‐reported airborne exposures were similar, but those with LVCR had longer average cumulative deployments (Table 2; 449.4 (±343.2) vs. 614.0 (±434.3) days, *p* = 0.009, *d* = 0.42). In the adjusted model investigating the association between deployment length and LVCR, the total months of deployment significantly increased the odds of having LVCR (aOR = 1.04, 95% CI [1.01, 1.07]).

### 3.4 Cardiopulmonary exercise testing and pulmonary function testing

**FIGURE 2:**
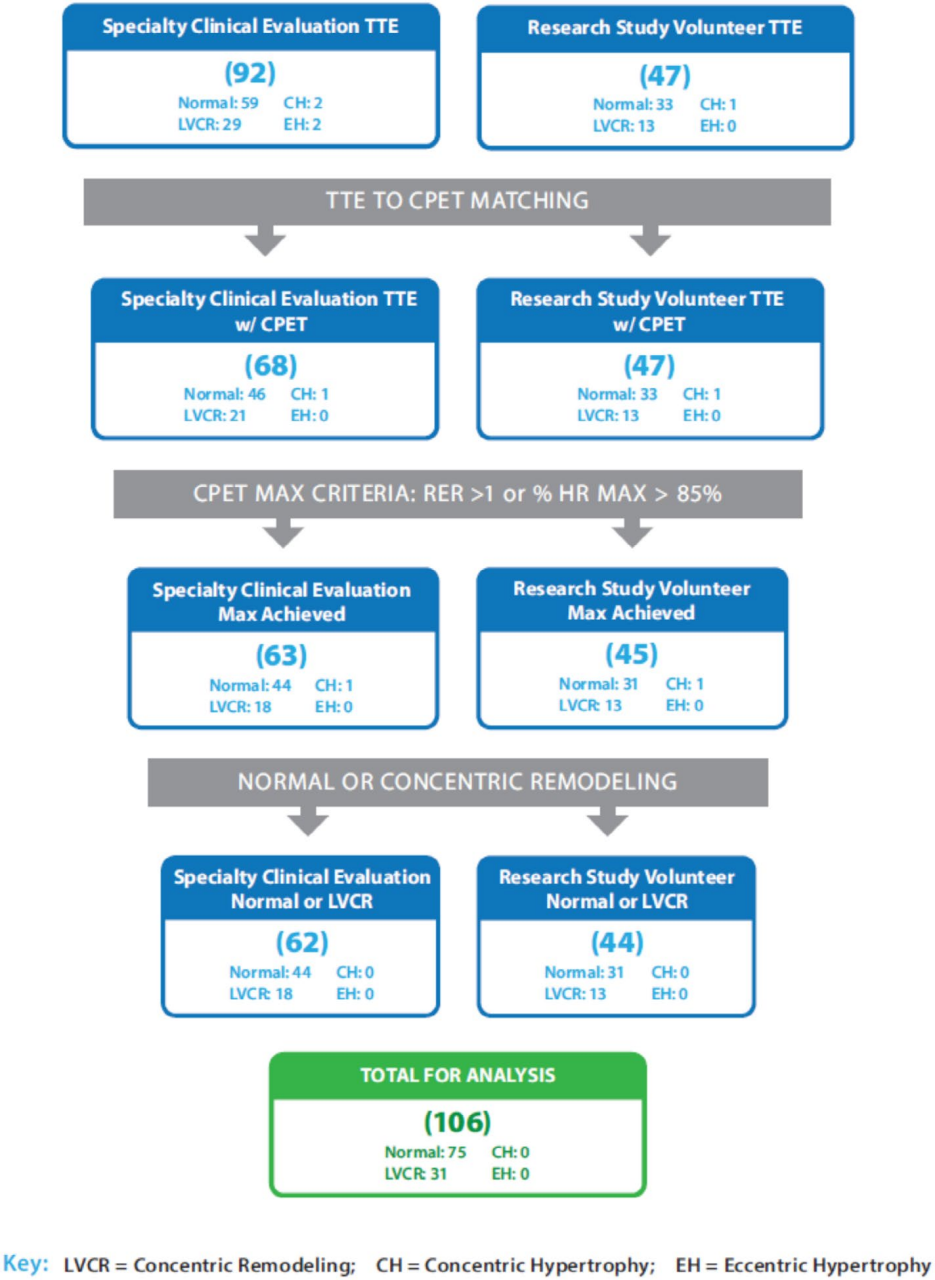
Flow diagram of the CPET filtering workflow, from the initial complete participant sample to those included in the CPET analysis, separated by participant cohort (Specialty Clinical Evaluation vs. Research Study Volunteer). CH, concentric hypertrophy; EH, eccentric hypertrophy; LVCR, left ventricular concentric remodeling; RER, respiratory exchange ratio; TTE, transthoracic echocardiogram.

CPET data were available for 113 of 134 veterans, with 106 achieving maximal effort, as illustrated in Figure 2. Unadjusted comparison of CPET performance between the normal and LVCR groups is shown in Table 3. Exercise capacity (V̇O_2_, mL/kg/min) was reduced on average (−9.5%; *d* = 0.41) and ventilation was less efficient (V̇_E_/V̇CO_2_ nadir: +6.6%; *d* = 0.56) among veterans with LVCR in comparison to those with normal LV geometry. The heart rate response to exercise was also blunted in those with LVCR relative to normal LV geometry, as evidenced by a reduced chronotropic index (−8.6%), reduced peak predicted heart rate (−5.0%), and increased heart rate reserve (+42.4%) with small‐to‐moderate effect sizes (*d* = 0.31 to 0.55).

**TABLE 3:** Cardiopulmonary exercise test (CPET) parameters for veterans with and without LV concentric remodeling (LVCR).

	Normal (*n* = 75)			LVCR (*n* = 31)			*p* Value	*d* Value
	*n*	Mean	SD	*n*	Mean	SD		
V̇O_2_ max (mL/min)	75	2372.83	634.00	31	2210.26	645.91	0.28	0.23
V̇O_2_/kg max (mL/kg/min)	75	26.18	6.65	31	23.68	5.31	0.04*	0.41
V̇O_2_/kg max pp	74	80.28	16.68	31	78.05	15.65	0.55	0.13
V̇E/V̇CO_2_ nadir	75	25.12	3.15	31	26.80	3.72	0.02*	0.48
O_2_ Pulse (mL/beat)	75	14.82	3.93	31	14.58	3.79	0.42	0.04
RER	75	1.20	0.12	31	1.20	0.14	0.91	0.03
Chronotropic response	75	1.16	0.32	31	1.06	0.31	0.10	0.32
HR max pp	74	91.10	6.82	31	86.50	9.50	0.02*	0.55
HR reserve (bpm)	75	17.33	11.80	31	24.73	15.69	0.02*	0.53
Δ pulse pressure (mmHg)	68	0.62	0.13	28	26.21	32.69	0.67	0.09
Δ diastolic pressure (mmHg)	69	28.68	34.37	29	7.55	12.85	0.40	0.17
Systolic BP (mmHg)	74	155.38	27.99	31	157.03	22.11	0.71	0.07
Diastolic BP (mmHg)	74	86.12	17.46	31	87.68	16.11	0.71	0.10
Power (Watts)^a^	57	186.84	41.53	25	175.60	46.00	0.30	0.25

*Note*: CPET parameters denote value at peak exercise unless otherwise stated. Δ Pressures are the difference from rest to peak. *p* and *d* values were determined by *t*‐tests and Cohen's *d* with Hedges correction, respectively.

Abbreviation: pp, percent predicted.

^a^ Cycle ergometer only (*n* = 82).

* Denotes statistical significance (*p* < 0.05).

**FIGURE 3:**
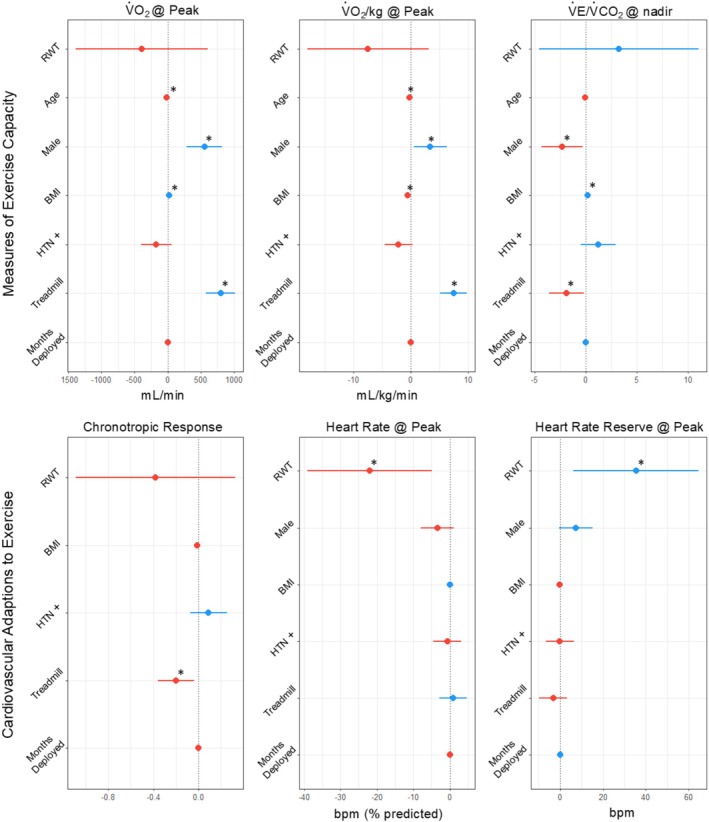
Results of multivariate linear regression model for select CPET variables. Each variable on the Y axis represents a covariate in each model. Red and blue dot and line plots represent the β coefficient (center dot) and the 95% confidence intervals (lines), with red denoting a negative β and blue denoting a positive β. *Represents a statistically significant association (*p* < 0.05) of the marked covariate with the respective CPET parameter. Units for each CPET parameter listed when present.

The results of the multivariate linear regression model are depicted in Figure 3. In the fully adjusted model, only achieved age‐predicted maximal HR and HR reserve at peak exercise were significantly associated with RWT. Additionally, an adjusted means analysis comparing the veterans with and without LVCR found a significant difference only between achieved age‐predicted maximal HR and HR reserve at peak when correcting for other parameters (Table 4).

**TABLE 4:** Mean differences in select CPET parameters between veterans with and without LVCR, adjusted for age, sex, BMI, exercise modality, and presence of hypertension.

	Adj mean diff	*p* Value
V̇O_2_ max (mL/min)	42.50	0.65
V̇O_2_/kg max (mL/kg/min)	1.01	0.33
V̇O_2_/kg max pp	1.40	0.67
V̇E/ V̇CO_2_ Nadir	−1.38	0.05*
O_2_ pulse (mL/beat)	−0.44	0.48
RER	0.01	0.67
Chronotropic response	0.11	0.11
HR max pp	4.42	0.01*
HR reserve (bpm)	−7.48	0.01*
Δ pulse pressure (mmHg)	0.03	0.18
Δ diastolic BP (mmHg)	−1.87	0.80
Systolic BP (mmHg)	−0.68	0.82
Diastolic BP (mmHg)	−3.17	0.56
Power (Watts)	3.57	0.68

* Denotes statistical significance (*p* < 0.05).

PFT data were available for 129 of 134 veterans. Spirometry, lung volume, and diffusion were similar between veterans with LVCR and normal LV geometry (Table 5). There were no significant differences in any of the percent predicted lung volumes or ratios. Finally, D_LCO_ did not differ between groups.

**TABLE 5:** Pulmonary function test (PFT) parameters for veterans with and without LV concentric remodeling (LVCR).

	Normal (*n* = 90)			LVCR (*n* = 39)			*p* Value	*d* Value
	*n*	Mean	SD	*n*	Mean	SD		
FEV1	90	3.75	0.87	39	3.46	0.90	0.09	0.33
FEV1 pp	88	96.98	16.84	39	92.38	19.82	0.21	0.25
FVC (L)	90	4.78	1.02	39	4.33	1.13	0.04*	0.42
FVC pp	88	99.33	14.38	39	93.16	18.66	0.07	0.37
FEV1/FVC	90	78.38	7.22	39	79.85	5.38	0.40	0.23
FEV1/FVC pp	88	97.09	8.69	39	99.24	7.81	0.27	0.26
TLC (L)	89	6.56	1.38	38	6.00	1.16	0.02*	0.44
TLC pp	87	94.43	15.28	38	89.44	13.99	0.08	0.34
RV (L)	89	1.69	0.87	38	1.59	0.61	0.45	0.14
RV pp	87	96.99	44.90	38	93.11	30.94	0.58	0.10
RV/TLC	89	25.03	10.29	38	26.98	10.00	0.32	0.19
RV/TLV pp	87	101.09	38.31	38	105.76	32.79	0.49	0.13
DLCO pp	87	95.23	14.56	38	93.40	16.08	0.55	0.12

*Note*: *p* and *d* values were determined by *t*‐tests and Cohen's *d* with Hedges correction, respectively.

Abbreviation: pp, percent predicted.

* Denotes statistical significance (*p* < 0.05).

## 4 DISCUSSION

In our sample of veterans deployed to the Southwest Asia Theater of Military Operations, approximately one‐third had evidence of left ventricular (LV) remodeling, specifically LV concentric remodeling (LVCR), as assessed by 2D echocardiography. Those with LVCR were deployed, on average, 5.5 months longer than those with normal LV geometry. The rates of remodeling found are markedly higher than those reported in a large civilian cohort with known risk factors (i.e., hypertension and diabetes mellitus) for cardiac remodeling (Figure 1). The presence of LVCR in both veterans from clinical specialty programs and research volunteers suggests it may be common in previously deployed individuals. The association between LV remodeling and reduced exercise performance redemonstrated here highlights the importance of considering non‐pulmonary contributions to exertional dyspnea in deployed veterans with military environmental exposures.

LV remodeling is a pathological, adaptive myocardial process that includes cardiomyocyte hypertrophy, apoptosis, and excess collagen deposition leading to interstitial myocardial fibrosis (Konstam et al., 2011). This process has been described as a generalized adaptive response to increased wall stress, mediated by alterations in the renin‐angiotensin‐aldosterone system, sympathetic nervous system, systemic inflammation, and oxidative stress, ultimately creating a thicker LV wall and a resultant decrease in LV chamber size. These physiologic changes are thought to mirror subclinical compensatory changes that are typically unaccompanied by symptoms, allowing for preservation of LV systolic function. LV remodeling can be assessed with several different imaging techniques, of which echocardiography remains the most widely utilized. The three patterns of LV remodeling determined by two‐dimensional echocardiography—concentric remodeling, concentric hypertrophy, and eccentric hypertrophy—were explored initially in the VALIANT study and their definitions have become universally accepted (Velazquez et al., 2003). Regardless of pattern, this remodeling has been proposed as an intermediate step towards the development of heart failure, with possible far‐reaching systemic consequences and associations with pulmonary airflow obstruction, chronic renal disease, anemia, and systemic inflammation (Cheng & Vasan, 2011).

In this study, veterans with LVCR were found to have reduced exercise capacity (V̇O_2_ peak) compared to those without remodeling, consistent with prior studies of abnormal LV geometry and exercise (Lam et al., 2010). Studies of patients with LV remodeling suggest that V̇O_2_ peak is linked to a reduction in LV end‐diastolic volume, as the LV chamber decreases in size as the remodeled walls thicken (Letnes et al., 2023). In keeping with LVCR as a precursor state for overt diastolic dysfunction, we also observed a trend towards increased LAVI in the group with LVCR (*p* = 0.08), reflecting remodeling of the left atrium associated with the chronically elevated filling pressures seen in the setting of impaired LV relaxation (Pritchett et al., 2005). During exercise testing, the LVCR group had a higher V̇E/V̇CO_2_ nadir than the normal group; this value is commonly used to determine the presence of ventilatory inefficiency, with higher values correlated with worsened outcomes in heart failure patients (Nayor et al., 2020). Additionally, chronotropic incompetence was suggested in the LVCR group, with higher heart rate reserve and lower heart rates based on percent predicted values at peak exercise. These findings mirror the chronotropic incompetence described in patients with increased LV mass and cavity size in the Framingham Study cohort, in which LV mass above the 90th percentile was associated with failure to achieve target predicted heart rates (Lauer et al., 1999). Finally, despite the known association between LV remodeling and obstructive lung disease and the nature of the inhaled toxins implicated, there was no suggestion of an increase in spirometric obstruction. In fact, percent‐predicted values for FVC, TLC, and RV were all significantly reduced in the LVCR group, suggesting a tendency towards restriction rather than obstruction that may reflect the slightly elevated BMI in the LVCR group.

There are several possible factors that may underpin the higher rates of pathological LV concentric remodeling observed among deployed veterans in our study. Cardiac remodeling is influenced by the aging process and by the presence of systemic hypertension, sleep apnea, diabetes mellitus, and obesity (Cheng & Vasan, 2011), all of which, save diabetes mellitus, were relatively frequent comorbidities amongst our veteran cohort. Notably, rates of obesity were higher in the veterans who sought evaluation for unexplained dyspnea. Accounting for these comorbidities, prevalence rates for remodeling were higher than would have been expected, and both veterans seen in specialty evaluation programs and volunteers were relatively young to develop a cardiac process typically associated with “normal aging.” We additionally sought to determine if differences in medication use could account for some of these differences; the frequency of the most common category of medication, statins, was not different between those with and without LVCR (11/92 vs. 8/42, *p* = 0.275), and use of other medications was less frequent. Aside from comorbidities associated with the metabolic syndrome, common to the entire cohort is a history of frequent exposure to airborne hazards during overseas military service. Air pollution has been demonstrated as a causal factor behind the development of cardiovascular disease in large civilian cohorts (Alexeeff et al., 2023; Brook et al., 2010). Exposure to a wide range of inhaled pollutants is a frequent and pervasive occurrence for veterans deployed to warzones in the Southeast Asia Theater of Military Operations. Common exposures during deployment include local ambient air pollution, engine exhaust, dust storms, and, perhaps most prominently, smoke generated from burn pits used for waste disposal (Garshick & Blanc, 2023). A greater total length of deployment, which may imply a greater cumulative duration of exposure to these inhaled pollutants, conferred a greater risk of developing LVCR in our study. When deployment time was evaluated as an independent variable in our multivariable regression model, it was found that the risk of LVCR increased by 4% for each month of deployment.

Burn pits are used to dispose of a variety of wastes, including chemicals, medical and human waste, metals, rubber, wood, food waste, and petroleum and plastic products (*Military Medicine*, 2015). Specific toxins identified from the burning of these wastes include particulate matter (PM), airborne metal particles, benzene, polychlorinated dibenzo‐p‐dioxins and dibenzo‐p‐furans (PCDDs/Fs), polycyclic aromatic hydrocarbons (PAHs), and volatile organic compounds (VOCs) (Masiol et al., 2016). There is evidence for each of these categories of pollutants to suggest cardiotoxicity in vitro and some evidence from population‐based studies to suggest cardiotoxic effects in humans. Benzene exposure raises circulating levels of angiogenic cells (Kutikhin et al., 2018), which may indicate endothelial stress, and increases inflammation and cell–cell adhesion in the myocardium of a mouse model of hypertension‐induced heart failure (Abplanalp et al., 2017; Zelko et al., 2021). Dioxin‐like compounds, a specific class of PAHs, have been shown as far back as 2001 to produce direct cardiovascular toxicity in animal models (Humblet et al., 2008; Jokinen et al., 2003; Lind et al., 2004), with chronic exposure producing increases in systemic blood pressure, circulating cholesterol and triglycerides, and resulting in chronic arteritis, myocardial hypertrophy, and cardiomyopathy (Marris et al., 2020). These effects were found to be linked to increases in inflammation, oxidative stress, and possible mitochondrial dysfunction (Biswas et al., 2008). Finally, smoke from combustion of plastic waste has been shown to produce higher amounts of systemic inflammation than nonplastic‐containing samples (Kim et al., 2021). A recent study of the effects of inhalation of micro‐ and nanoparticles (MNPs) of polyamide powder (i.e., nylon, ubiquitous in military supply chains) in an animal model showed systemic inflammation, increased blood pressure, and impaired vascular dilatation, while no increases in inflammation were seen in the lung vasculature (Cary et al., 2023). The cardiotoxic effects of MNPs have been examined more directly in humans with the finding that microplastic and nanoplastic content in carotid artery plaques conferred a higher risk of myocardial infarction, stroke, or death (Marfella et al., 2024). Both translational studies to demonstrate that the cardiotoxic effects in animal models have analogous effects in humans as well as larger population‐based studies will be critical to establish a connection between inhalation of the toxic compounds emanated by burn pits and the development of cardiotoxic effects, and to determine the factors underlying differential development of cardiotoxicity between participants with similar exposure patterns.

Beyond the effects of specific pollutants, the effects of both the duration and intensity of air pollutant exposure on cardiovascular disease have been the subject of intense investigation. Both long duration, low intensity exposure, such as that encountered in urban and traffic associated pollution (Aung et al., 2018), or short duration, high intensity exposure, like that experienced by disaster relief workers, have been shown to result in adverse concentric remodeling (Pope et al., 2011). In older individuals, short duration, low intensity exposure can also greatly increase the risk of developing cardiovascular disease (Gestro et al., 2020). Military veterans deployed to Southwest Asia Theater of Military Operations represent a unique population exposed to a wide range of air pollutants at intense levels; in these countries, reported PM_2.5_ levels have exceeded 10,000 μg/m^3^ (Falvo et al., 2015), far above the 25 μg/m^3^ level set as the allowable daily exposure by the World Health Organization. This high level of PM is derived from both geologic sources (e.g., dust storms) and anthropogenic sources, including industrial pollution, civilian and military combustion, and burn pits (Engelbrecht, McDonald, & Gillies, 2009; *Military Medicine*, 2015). Due to the average deployment length of 15.5 (±10.9) months, and with many individuals having multiple deployments, veterans experience a unique pattern of exposure to inhaled pollutants characterized by moderate duration and high intensity. Possible long‐term physiological effects of the exposures may be exacerbated by the unique characteristics of combat experience that can make an individual more susceptible to the effects of air pollution, such as high stress (McFarlane, 2010), high physical demands (DeFlorio‐Barker et al., 2020), and the requirements of certain military occupational specialties (Zell‐Baran et al., 2019), all of which may influence the development of systemic hypertension (Cai et al., 2016; Howard et al., 2020). Our observation that longer deployment length was associated with LVCR may represent a composite of all of these considerations. Cardiovascular outcomes and rates of concentric remodeling from such moderate duration, high intensity exposures up to this point have not been well‐demonstrated.

This study has several notable strengths and limitations. A strength of this study is the inclusion of two separate deployed veteran cohorts—that is, those seen in specialty evaluation programs and research volunteers—who were evaluated nationally across five separate VA medical centers. In the absence of an available control cohort, the research volunteer cohort served as an a priori control group for comparison purposes. Several limitations should be acknowledged regarding available data. Our cohort was predominantly male, limiting the ability to determine sex differences in our results, and the veterans evaluated in the specialty cohort were significantly older than the research volunteer cohort. Exercise testing and echocardiographic data were incomplete or missing for some veterans, in part due to variations in reporting across centers, and CPET modality and protocols chosen were not universal, though the majority were performed using the same cycle ergometry protocol. Available rates of LV remodeling in the general population are sparse, and comparisons of our veteran cohort with a large civilian cohort should be interpreted with caution, as our veteran cohort was younger and predominantly male. However, given the strong association of LV remodeling with increasing age (Cheng et al., 2009), this may serve to strengthen our conclusions. Finally, quantified assessment of airborne hazard exposure during military service is not available, with exposure intensity and duration determined secondarily by deployment length and veteran self‐report.

## 5 CONCLUSIONS

In this study of veterans formerly deployed to the Southwest Asia Theater of Military Operations, rates of LV remodeling were markedly higher than historical rates established by a large population‐based study, the Framingham Heart Study. This elevated prevalence was found to be the case both for veterans who presented to specialized clinical evaluation centers for unexplained dyspnea and those who were not seeking treatment. The presence of LV remodeling was associated with multiple abnormalities during maximal cardiopulmonary exercise testing, including a significant reduction in exercise capacity, ventilatory efficiency, and heart rate response to exercise. The rates of LV remodeling may have been influenced to a modest degree by relatively high rates of obesity but does not fully explain our findings. More research is required to determine if specific airborne hazard exposures represent a likely mediator for the development of this pathophysiological change to the myocardium.

## FUNDING INFORMATION

This work was supported by Merit Review Award # I01 CX001515 from the United States (U.S.) Department of Veterans Affairs Clinical Sciences Research and Development Service and supported in part by the Airborne Hazards and Burn Pits Center of Excellence and. The contents do not represent the views of the U.S. Department of Veterans Affairs or the United States Government.

## ETHICS STATEMENT

Ethical approval for this study was provided by the VA New Jersey Health Care System's Institutional Review Board (IRB) and Research and Development Committee. Research participants provided written consent prior to participating. The authors report no relevant conflicts of interest.

## Supporting information


Appendix S1.
