# Supplementary material for: Left ventricular concentric remodeling is highly common among veterans deployed to Southwest Asia and is associated with impaired exercise performance
Source: Physiol Rep. 2025 Jul 9;13(13):e70445. doi: 10.14814/phy2.70445 (PMC12238780; doi:10.14814/phy2.70445)
Supplement: Supplementary file 1 — Appendix S1. [file PHY2-13-e70445-s001.docx]

**Table S1:** Basic Demographic characteristics of the Research Study Volunteer Veterans, the Specialty Clinical Evaluation Veterans, the combined Veterans, and the comparative civilian population (von Jenison et al 2020). Data are presented as mean (SD) or presented as a percentage of the total within each subject cohort.

|  | Research Study Volunteer | Specialty Clinical Evaluation | Combined Veteran | Framingham |
| --- | --- | --- | --- | --- |
|  | (n = 47) | (n = 92) | (n=139) | (n=5741) |
| Age | 40.36 (7.78) | 46.27 (9.49) | 44.27 (9.35) | 50 (15) |
| Sex (Female) | 10.64% | 15.22% | 13.67% | 54.6% |
| **Race** |  |  |  |  |
| White | 87.23% | 77.53% | 80.88% | ~95%^A^ |
| Black | 10.64% | 11.24% | 11.03% | <5 %^A^ |
| Asian | 2.13% | 2.25% | 2.21% | <5 %^A^ |
| Other/Multiple | 0.00% | 8.99% | 5.88% | <5 %^A^ |
| **BMI** | 28.32 (3.44) | 31.45 (5.81) | 30.39 (5.33) | 27 (5.1) |
| Normal (%) | 19.15% | 8.70% | 12.20% | 38.80% |
| Overweight (%) | 55.32% | 39.13% | 44.60% | 38.10% |
| Obese (%) | 25.53% | 52.17% | 43.20% | 23.10% |
| Hypertension (%) | 8.51% | 36.96% | 27.34% | 26.10% |
| **Smoking** |  |  |  |  |
| Active (%) | 2.13% | 1.22% | 1.55% | 17% |
| Former (%) | 29.79% | 32.93% | 31.78% | 14% |
| Never (%) | 68.09% | 65.85% | 66.67% | 70% |
| **Echo** |  |  |  |  |
| RWT | 0.36 (0.10) | 0.40 (0.09) | 0.39 (0.10) | 0.38 (0.05) |
| LVMI (g/m^2^) | 69.57 (20.29) | 71.63 (19.18) | 70.93 (19.52) | 84.2 (17.5) |
| Lat E/e' | 5.80 (1.52) | 6.98 (2.84) | 6.47 (2.43) | 6.3 (1.9) |
| Systolic Pressure (mmHg) | 115.00 (12.2) | 124.00 (12.00) | 120.02 (12.78) | 121 (16) |
| Diastolic Pressure (mmHg) | 73.20 (9.17) | 86.00 (14.30) | 80.95 (13.94) | 74 (10) |

1. Ethnicity data for the FHS cohort was estimated from Von Jenison et al (n = 7414), which utilized data from the Offspring, Generation, and Omni 1 Cohorts

| **A.**  Research Study Volunteer | Normal | Concentric Remodeling | Concentric Hypertrophy | Eccentric Hypertrophy | Totals | % of Total With LVCR |
| --- | --- | --- | --- | --- | --- | --- |
| **BMI** |  |  |  |  |  |  |
| Normal | 8 | 1 | 0 | 0 | **9** | 11.11% |
| Overweight | 18 | 8 | 0 | 0 | **26** | 30.77% |
| Obese | 7 | 4 | 1 | 0 | **12** | 33.33% |
| Totals | **33** | **13** | **1** | **0** | **47** | 27.66% |
| **HTN** |  |  |  |  |  |  |
| Yes | 3 | 1 | 0 | 0 | **4** | 25.00% |
| No | 30 | 12 | 1 | 0 | **43** | 27.91% |
| Totals | **33** | **13** | **1** | **0** | **47** | 27.66% |
|  |  |  |  |  |  |  |
|  |  |  |  |  |  |  |
| **B.**  Specialty Clinical Evaluation | Normal | Concentric Remodeling | Concentric Hypertrophy | Eccentric Hypertrophy | Totals | % of Total With LVCR |
| **BMI** |  |  |  |  |  |  |
| Normal | 7 | 1 | 0 | 0 | **8** | 12.50% |
| Overweight | 23 | 13 | 0 | 0 | **36** | 36.11% |
| Obese | 29 | 15 | 2 | 2 | **48** | 31.25% |
| Totals | **59** | **29** | **2** | **2** | **92** | 31.52% |
| **HTN** |  |  |  |  |  |  |
| Yes | 19 | 11 | 2 | 2 | **34** | 32.35% |
| No | 40 | 18 | 0 | 0 | **58** | 31.03% |
| Totals | **59** | **29** | **2** | **2** | **92** | 31.52% |
|  |  |  |  |  |  |  |
|  |  |  |  |  |  |  |
| **C.** Combined | Normal | Concentric Remodeling | Concentric Hypertrophy | Eccentric Hypertrophy | Totals | % of Total With LVCR |
| **BMI** |  |  |  |  |  |  |
| Normal | 15 | 2 | 0 | 0 | **17** | 11.76% |
| Overweight | 41 | 21 | 0 | 0 | **62** | 33.87% |
| Obese | 36 | 19 | 3 | 2 | **60** | 31.67% |
| Totals | **92** | **42** | **3** | **2** | **139** | 30.22% |
| **HTN** |  |  |  |  |  |  |
| Yes | 22 | 12 | 2 | 2 | **38** | 31.58% |
| No | 70 | 30 | 1 | 0 | **101** | 29.70% |
| Totals | **92** | **42** | **3** | **2** | **139** | 30.22% |

**Table S2:** Rates of Left Ventricular Remodeling in the study sample; A) Research Study Volunteers, B) Specialty Clinical Evaluations, and C) Combined Veteran Sample. Rates are stratified by Body Mass Index (BMI) class, or by the presence of Hypertension (HTN). Rate of LVCR corresponds to the total of each row.

SHADE Survey Details

The SHADE survey is an investigator-led questionnaire to quantify five domains of inhalational exposures: 1) Military Occupational-Related Vapors, Gas, Dust or Fumes (VGDF), 2) burn pit smoke, 3) open air combustion byproducts (non-burn pit), 4) combustion engine exhaust and dust exposure (mechanical and natural sources), and 5) toxicants, on a 0-100 scale based upon duration and intensity. For this study, exposure domain scores were binned into three categories relative to published median values from SHADE’s population-based sample:: i) high (> median), ii) low (≤ median), or no exposure (0).^24^

77 of the specialty clinical evaluation veterans completed the SHADE questionnaire. Those with LVCR had a lower Open-Air Combustion (other than burn pits) and toxicants exposure score compared to those with a normal LV. In the adjusted logistic regression models, both Open Air Combustion exposure (other than burn pits) and toxicants were associated with a reduced odds of having LVCR (aOR = 0.969, 95% CI [0.946, 0.989] and aOR = 0.972, 95% CI [0.944, 0.997], respectively). Complete results of the SHADE analysis can be found in table S3 below.

|  | ` |  | Normal (n = 51) | | | LVCR (n = 26) | | | p | d/V |
| --- | --- | --- | --- | --- | --- | --- | --- | --- | --- | --- |
| Score | | **Exposures (SHADE)** |  |  |  |  |  |  |  |  |
|  |  | VGDF | 51 | 19.39 (21.06) | | 26 | 13.68 (13.35) | | 0.15 | 0.32 |
|  |  | Burn Pits | 51 | 66.67 (27.23) | | 26 | 63.94 (22.73) | | 0.64 | 0.11 |
|  |  | Other Open Air | 51 | 44.36 (26.02) | | 26 | 24.52 (26.8) | | <0.005 | 0.74 |
|  |  | Vehicle/Dust | 51 | 50.25 (19.16) | | 26 | 47.12 (22.17) | | 0.54 | 0.15 |
|  |  | Toxicants | 51 | 24.18 (22.44) | | 26 | 14.74 (20.72) | | 0.07 | 0.43 |
| Exposure Level | | **VGDF** | **51** |  |  | **26** |  |  |  |  |
|  |  | None | 13 | 25.49% | | 6 | 23.08% | | 0.299 | 0.177 |
|  |  | Low | 13 | 25.49% | | 11 | 42.31% | |  |  |
|  |  | High | 25 | 49.02% | | 9 | 34.62% | |  |  |
|  |  | **Burn Pits** | **51** |  |  | **26** |  |  |  |  |
|  |  | None | 0 | 0.00% | | 0 | 0.00% | | 0.424 | 0.091 |
|  |  | Low | 25 | 49.02% | | 16 | 61.54% | |  |  |
|  |  | High | 26 | 50.98% | | 10 | 38.46% | |  |  |
|  |  | **Other** | **51** |  |  | **26** |  |  |  |  |
|  |  | None | 4 | 7.84% | | 11 | 42.31% | | <0.001 | 0.425 |
|  |  | Low | 21 | 41.18% | | 9 | 34.62% | |  |  |
|  |  | High | 26 | 50.98% | | 6 | 23.08% | |  |  |
|  |  | **Vehicle/Dust** | **51** |  |  | **26** |  |  |  |  |
|  |  | None | 0 | 0.00% | | 1 | 3.85% | | 0.342 | 0.167 |
|  |  | Low | 31 | 60.78% | | 14 | 53.85% | |  |  |
|  |  | High | 20 | 39.22% | | 11 | 42.31% | |  |  |
|  |  | **Toxicants** | **51** |  |  | **26** |  |  |  |  |
|  |  | None | 17 | 33.33% | | 14 | 53.85% | | <0.05 | 0.285 |
|  |  | Low | 7 | 13.73% | | 6 | 23.08% | |  |  |
|  |  | High | 27 | 52.94% | | 6 | 23.08% | |  |  |

**Table S3:** Exposure analysis with the Specialty Clinical Evaluation veterans (n =77). Scores range from 0 to 100 based on duration and intensity, with 0 denoting no exposure. Exposure levels were categorized as none, low or high, based upon the median exposure score in each domain for the whole sample, with “High” being greater, and “Low” being less but greater than 0. Continuous variables are presented as mean (SD). Categorical variables are presented as a percentage of the total within each subject cohort. p values and effect sizes were determined by t-tests (Wilcox as required) and Cohen’s d with Hedge’s correction for continuous variables and with chi-squared and Cramer’s V for categorical variables, respectively.
